# Schauta’s Clinic—Some Impressions of Interest in Obstetrics and Gynecology

**Published:** 1896-06

**Authors:** Electa B. Whipple

**Affiliations:** Buffalo, N. Y.; 491 Porter Avenue


### SCHAUTA'S CLINIC—SOME IMPRESSIONS OF INTEREST IN OBSTETRICS AND GYNECOLOGY.

BY ELECTA B. WHIPPLE, A. M., M. D., Buffalo, N. Y.

NOTWITHSTANDING the vast opportunities afforded by the clinics of London and other foreign cities, Vienna undoubtedly furnishes the greatest advantages to the general practitioner, as well as to the specialist in every department of medical science.

This is apparent from the large number of students of almost every nationality there congregated for scientific research. Another reason is the unlimited amount of clinical material, and also there are certain forms of disease quite prevalent in Austria and Hungary which the practitioner rarely meets in other localities.

Excellent courses in surgery, gross pathology, bacteriology, histology and general pathology may be had, while special advantages are offered in the throat, nose, eye, ear and skin clinics, and it is doubtful if as good courses in internal medicine can be found anywhere else in the world. Nor is the field in obstetrics less fruitful, since in the three clinics at the Allgemeines Krankenhaus —Schauta's, Chrobak's and Braun's—12,000 births are annually recorded and in Professor Schauta's clinic alone there are yearly 4,000 births. The post-graduate courses in diagnostic and operative obstetrics are many and good, and every phase from the normal, uncomplicated labor to the Cesarean section may be seen. The number of students admitted to a given course is limited—not more than from seven to ten being allowed in the same class.

In the maternity ward, patients in the latter months of pregnancy are daily presented for examination, while on "aufnahme," or reception days, clinics in every stage of labor are present. In all of these cases the external examination for correct diagnosis is regarded of paramount importance and abdominal palpation, with the use of the pelvimeter for recording exact measurements, is practised. In making internal examination, one or two—preferably two—fingers are employed. These examinations are conducted under the most rigid antiseptic precautions. The hands of the examiner must be cleansed with (*a*) soap and brush, (*b*) absolute alcohol, (*c*) sublimate solution, 1:1000. In this the requirements are most imperative and carelessness is in no degree tolerated. In Professor Leopold's clinic at Dresden, the directions for hand ablution are the same except a second 1:2000 bichloride solution is substituted for the absolute alcohol. In his *Grundriss der Operativen Geburtshülfe*, Professor Schauta advocates the use of alcohol as a disinfectant in obstetric practice because it exerts solvent properties upon the fatty tissues, thus allowing bichloride of mercury to come in immediate contact with the bacteria contained upon the hands.

In the *Deutschen Medicinische Wochenschrift*, 1896, No. 6, investigations conducted by Ahfield and Vahle are described. Ahfield found that alcohol does not act simply by dissolving fatty substances upon the hands, because other agents are more solvent though less antiseptic, but it exerts a direct influence in destroying virulent streptococci. Experiment was made with amniotic membrane to ascertain the influence of alcohol upon such tissues when wet and when dry. Alcohol acted readily when the membrane was moistened, but exerted only feeble influence upon dried tissues. Experiments upon the hands of attendants and nurses demonstrated the value of alcohol as a disinfectant.

An antiseptic vaginal douche is given every patient when labor begins, and as it advances the genitals are frequently bathed with a similar solution. As the head is passing over the perineum, the attendant, by giving it support, uses every precaution to prevent injury and yet the perineum is sometimes lacerated. A report of the obstetrical cases in Schauta's clinic from 1892 to 1894, published in the *Monatsschrift für Geburtshülfe und Gynekologie*, July, 1895, as given by his assistant, Dr. H. von Woerz, in regard to the occurrence of rupture of the perineum, shows several conditions that endanger the perineum by a too sudden passage of the head over it, viz.: (*a*) sudden surprise to the woman as the head is born ; (*b*) strong and sudden action of the uterus combined with that of the abdominal muscles when the head is passing the perineum ; (*c*) rapid delivery by operative interference ; (*d*) small head advancing rapidly and quickly dilating the vulva ; (*e*) deformed pelvis in which there may be a large outlet, through which the fetus passes quickly, or owing to an uncurved sacrum and narrowing of the pubic arch, the head tends to be directed more nearly against the sacral segment of the pelvic floor. He states that: (*a*) in labors the frequency of rupture of the perineum varies directly with the size of the born child; (*b*) ruptures are more common when the perineum is not guarded than when it is; (*c*) by carefully employed protection of the perineum, the ratio between the weight of the child and frequency or extent of the rupture may be considerably modified.

The placenta, in normal cases, is left for nature to expel. If in course of three hours this is unaccomplished, the accoucheur removes it by Crèdè's method.

In placenta previa the Braxton Hicks method is employed and an assistant at the bedside of the patient exercises the strictest surveillance, be it one or several hours until nature performs her work—with the reward of saving mother and child. This is done under thorough antisepsis, as are craniotomies, decapitations, Cesarean sections and all obstetrical operations. No examination of parturient patients is permitted the student within twenty-four hours after he has made one of a patient undergoing operation of fetal decapitation.

In Cesarean section the instruments and hands of assistants and operator are most carefully made aseptic. The operator, clad in large rubber boots and long duck coat, over which is a great rubber apron, sits upon a high stool while operating. The operation is quickly performed and after the different stages are completed, the abdominal wound is closed with superficial catgut and deep silver-wire sutures, secured with glass pressure plates and buttons. The dressing consists of iodoform gauze, a thick layer of carbolised cotton and over all a closely fitting bandage of wadding or unstarched gauze.

In gynecology, as in obstetrics, the material is abundant. Laparatomies are made with the greatest care in every detail. Plastic operations are also made under a thorough aseptic regime, and vaginal fixations are much practised in those cases of retropositions which require operative interference.

Besides the cases for operation there is a large clinic of outpatients who daily present themselves for local treatment, which consists of pelvic massage, vaginal irrigation, tamponage and electricity. Trained nurses are unknown in this clinic, as well as in every other, but here a coarse servant, taught to do the few offices required by the doctor, is present to answer his call and receive any fee with which the students feel inclined to reward her.

In the obstetrical clinic, however, trained midwives—graduates of the school of midwifery which is connected with Braun's clinic—are in constant attendance. In Professor Sänger's private clinic, at Leipsic, pelvic massage is practised and in the polyclinic, which is under his supervision, vaginal irrigation is not only used but the needy out-patients are supplied with irrigators and taught self-use of them. Patients in this polyclinic are shown the greatest kindness and consideration—a virtue which does not always prevail in the charity clinics on the Continent. It has been said, "In Europe, in the great hospitals, the patients are looked upon as material to be shaped and fashioned as the surgeon sees best, irrespective of the patient's feelings."

A feature which particularly impressed me, and, to their credit be it said, was characteristic of the foreign clinics in which I was a student, was the marked respect and profound reverence always accorded the honored chief. Thus, when Professor Schauta entered the clinic room, he was invariably escorted by two or more of his assistants and every student rose in his place and remained standing until the professor was seated. And this instance was not an exception to the universal rule.

491 Porter Avenue.

